# Efficacy and safety of mirabegron in the treatment of overactive bladder syndrome after radical prostatectomy: a prospective randomized controlled study

**DOI:** 10.3389/fonc.2023.1188619

**Published:** 2023-05-02

**Authors:** Wang Li, Yanduo Lin, Hong Xie, Qiang Fu, Rong Chen, Xiaoyong Hu, Jianwen Huang, Jihong Wang, Ranxing Yang

**Affiliations:** ^1^ Department of Urology, Shanghai Sixth People’s Hospital Affiliated to Shanghai Jiao Tong University School of Medicine, Shanghai Jiao Tong University, Shanghai, China; ^2^ Department of Urology, Hainan Hospital, General Hospital of the Chinese People's Liberation Army, Hainan, China

**Keywords:** mirabegron, overactive bladder syndrome, radical prostatectomy, urinary incontinence, nocturia

## Abstract

**Objectives:**

To evaluate the effects of mirabegron in the treatment of overactive bladder syndrome (OAB) after radical prostatectomy (RP).

**Patients and methods:**

A total of 108 post-operative RP patients were randomly assigned to either the mirabegron (study) or the placebo (control) group. The Overactive Bladder Syndrome Self-Assessment Scale (OABSS) was selected as the primary endpoint, and the International Prostate Symptom Score (IPSS) and Quality of Life (QOL) score were selected as secondary endpoints. Statistical analysis was performed using IBM SPSS Statistics 26, and the treatment effects were compared between the two groups using independent samples t-test.

**Results:**

In total, 55 patients were included in the study group and 53 patients in the control group. The mean age was(70.08 ± 7.54)years. There was no statistical difference in the baseline data between the two groups. OABSS scores decreased significantly in the study group compared to the control group during drug treatment (6.67 ± 1.06 vs. 9.14 ± 1.83, p < 0.01) and were better than the control group during the follow-up at week 8 and week 12. In addition, the decrease in IPSS scores (11.29 ± 3.89 and 15.34 ± 3.54, p<0.01) and the increase in QOL scores (2.40 ± 0.81 vs. 3.20 ± 1.00) were statistically significant in the study group. And the patients in the study group had better improvement in voiding symptoms and quality of life than the control group during the follow-up period.

**Conclusion:**

Daily administration of 50 mg mirabegron after RP surgery significantly improved the symptoms of OAB after surgery with fewer side effects. Additional randomized controlled trials should be conducted in the future to further evaluate the efficacy and safety of mirabegron.

## 1 Introduction

Prostate cancer affects millions of men annually, making it the second most prevalent cancer in men, following lung cancer. It accounts for 7.11% of new cancer diagnoses in men worldwide (1). Its treatment methods include nonsurgical and surgical treatment. The former consists mainly of androgen deprivation therapy (ADT), radiation therapy (RT), chemotherapy and emerging immunotherapy. However, laparoscopic radical prostatectomy (LRP) remains the most effective and commonly accepted procedure for localized prostate cancer (2). Despite significant improvements in surgical methods and techniques, patients who undergo LRP commonly experience overactive bladder syndrome (OAB), with studies indicating that the prevalence of OAB following radical prostatectomy (RP) can reach as high as 37.8% (3). As a result of surgical injury to anatomical areas such as the external urethral sphincter and urethral smooth muscle, patients may develop symptoms of overactive bladder (OAB) including urinary frequency, urgency, and urge incontinence. Moreover, damage to the nerves innervating the bladder during surgery can result in postoperative overactivity of the patient’s detrusor muscle (DO), which is a significant contributing factor to OAB (4, 5). To assess these symptoms comprehensively and reliably, the Overactive Bladder Syndrome Self-Evaluation Scale (OABSS) is a valuable questionnaire consisting of four questions: daytime urination frequency, nocturia, urgency, and incontinence. These questions provide an objective and effective evaluation of the patient’s postoperative bladder condition and recovery (6).

Anticholinergics (such as solifenacin and tolterodine) have traditionally been the preferred treatment for patients suffering from overactive bladder (OAB); however, these drugs are associated with various adverse effects, including constipation, dry mouth, and blurred vision, and may even cause urinary retention or increased postvoiding residue (PVR) (7). Consequently, patient compliance with these medications is often poor. In contrast, mirabegron, the first β3-adrenoceptor agonist approved for the treatment of OAB (8), has been shown to be more effective than anticholinergics as it promotes detrusor relaxation, increases bladder capacity, and reduces urination frequency (9). Despite its potential benefits, there is limited research and data available on the effectiveness of mirabegron in treating OAB following LRP. Therefore, the primary objective of this article is to evaluate the safety and efficacy of mirabegron in managing lower urinary tract symptoms after LRP.

## 2 Materials and methods

### 2.1 Study participants

In this study, 116 consecutive patients who underwent PR between July 2020 and July 2022 in the Department of Urology of the Sixth People’s Hospital of Shanghai Jiao Tong University and had postoperative urinary abnormalities of varying degrees were recruited. Inclusion criteria included: (і) preoperative patients all had prostate cancer confirmed by ultrasound-guided trans-perineal prostate puncture and postoperative pathology showed limited prostate cancer ((clinical stage from T1c to T3a, N0 and M0)); (іі) symptoms such as urinary frequency, urgency, and urinary incontinence occurred after the urinary catheter was removed; and (ііі)patients were not taking drugs affecting bladder function before surgery (8). Exclusion criteria included: (і) patients who had received neoadjuvant chemotherapy or radiotherapy for prostate cancer, had neurogenic bladder, urinary tract infection, arrhythmia symptoms, or any history of surgery on urethra, bladder neck, or prostate in the past 3 months; (іі) patients with previous impaired liver function (Child-Pugh classification C or worse) or severe renal dysfunction (creatinine clearance < 30 mL/min) and (ііі) patients with moderate and severe OAB before surgery (OABSS total score > 5).

### 2.2 Research design

This is a prospective randomized, controlled study that was approved by the Ethics Committee of the Sixth People’s Hospital of Shanghai Jiao Tong University with approval number 2020-K-108(K) prior to the start of this study. All patients provided written informed consent and completed the IPSS and OABSS evaluations before surgery. After completion of relevant preoperative investigations, the patients all underwent transperitoneal laparoscopic radical prostatectomy for prostate cancer. During the operation, a pneumoperitoneum was established with the retropubic space. The main points of the operation included: (і) clearing the pelvic lymph nodes to observe whether the patient had tumor metastasis; (іі) suturing to cut the deep dorsal venous complex; (ііі) to maximize the removal of prostate tissue, we used extrafascial resection for all patients, while also sacrificing the neurovascular bundle; (IV) separating the seminal vesicles and the dorsal aspect of the prostate; (V) dissecting the lateral prostatic ligament and the bladder neck, (VI) dissecting and reconstructing the urethra. Postoperatively, a drainage tube is placed behind the pubic bone and an F22 catheter is left in the bladder.

Patients were randomly assigned to the study or control group through a computer-generated randomization procedure. All patients began Kegel exercise to train pelvic floor muscle function the day after surgery as directed by the treatment team. On this basis, patients in the study group were administered oral mirabegron sustained-release tablets daily (50 mg; Astellas Pharmaceutical Technologies Inc., Sinopharm J20180019; once a day); patients in the control group were given oral placebo (vitamin C, 50 mg) daily. Both study and control drugs were white round tablets in appearance and were treated for 1 month. Patients were discharged after drainage tube was removed; criteria for drainage tube removal were the absence of infection and anastomotic leakage, with drainage tube fluid < 20 ml for 2 consecutive days. Approximately 10-14 days after surgery, the patient was followed up in the urology clinic to have the urinary catheter removed, and the PSA value and the maximum urine flow rate Qmax re-evaluated. Weekly telephone follow-up was performed during the medication period and continued for 2 months after the treatment period, comparing the OABSS, IPSS, QOL scores, and the number and duration of bladder spasms during spontaneous voiding in both groups and using a visual analog scale (VAS) to measure the severity of bladder spasm.

### 2.3 Statistical analysis

All data were processed by SPSS v.26.0 software for Windows (IBM, Amonck, New York, USA), and continuous variables with normal distributions were represented as the mean ± standard deviation (SD). Independent sample t tests were used to determine differences in patient demographics, follow-up time, and outcomes during treatment between the two groups. The chi-square test was used to compare other clinical features between the two groups. P < 0.05 was considered statistically significant.

## 3 Results

### 3.1 Demographic and baseline characteristics

**Figure 1:**
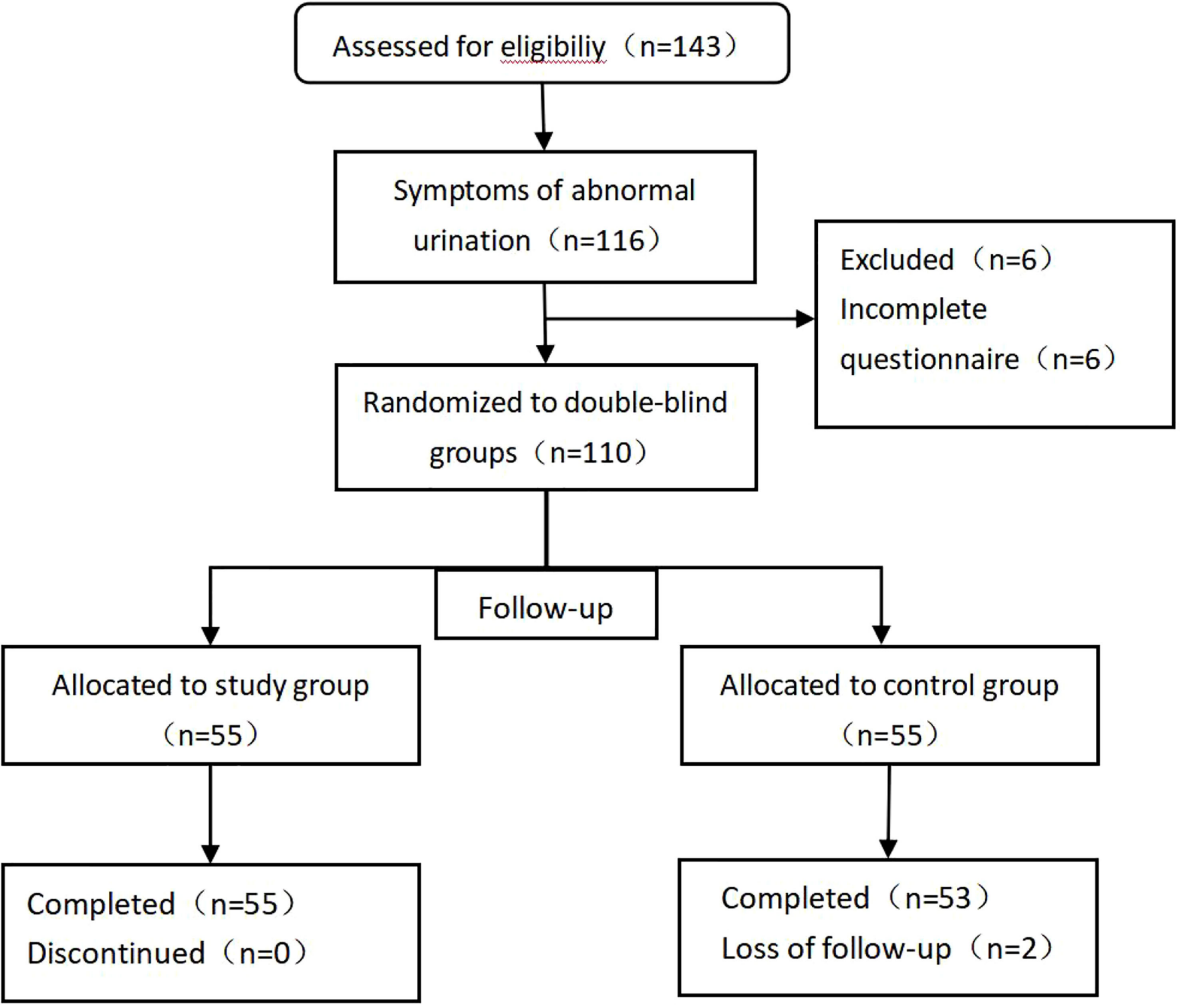
Flowchart for patients selection.

The study originally enrolled a total of 116 patients with abnormal urination after prostate cancer surgery; during follow-up, 6 patients did not complete the questionnaire, and 110 patients eventually completed the trial questionnaire. A total of 110 patients were randomly assigned to the study group (n=55) and the control group (n=55). During the follow-up, 2 individuals in the control group were excluded by interrupting the follow-up (**Figure 1**). The mean age was (70.08 ± 7.54) years. The general data of the two groups are shown in **Table 1**. There were no statistically significant differences in age, body mass index, PSA, prostate volume, operative time, intraoperative bleeding, postoperative HB reduction values, number of days in hospital,preoperative OAB scores, IPSS scores, and QOL scores between the two groups.

**Table 1:** Population demographics and clinical characteristics of the two groups of patients; preoperative patient OABSS, IPSS, QOL scores and postoperative prostate tumor pathological staging.

Variables, mean ± SD	Study group (n = 55)	Control group (n = 53)	P value
Age, year	70.84 ± 8.08	69.25 ± 6.90	0.28
BMI, kg/m2	25.36 ± 3.22	24.91 ± 2.83	0.32
Prostate-specific antigen, ng/ml	21.66 ± 23.74	17.46 ± 12.88	0.25
Prostate volume, ml	52.18 ± 25.78	47.32 ± 24.22	0.23
Operation time, min	170.18 ± 54.16	163.0 ± 64.9	0.85
Intraoperative bleeding, ml	214.69 ± 194.94	214.8 ± 202.4	1.00
Hemoglobin decrease value, g/L	14.87 ± 7.25	17.37 ± 9.93	0.14
Number of days in hospital	9.80 ± 3.21	9.16 ± 2.98	0.29
Preoperative index			
OABSS score	4.22 ± 1.18	4.29 ± 1.27	0.75
IPSS score	20.27 ± 4.36	19.37 ± 3.24	0.23
QOL score	3.27 ± 1.10	3.25 ± 1.16	0.94
Postoperative pathological stage			
< 2b	7(0.13)	3(0.06)	
2c	33(0.60)	34(0.64)	
3a	8(0.15)	8(0.15)	
3b	7(0.13)	8(0.15)	

### 3.2 Effect comparison

**Figure 2:**
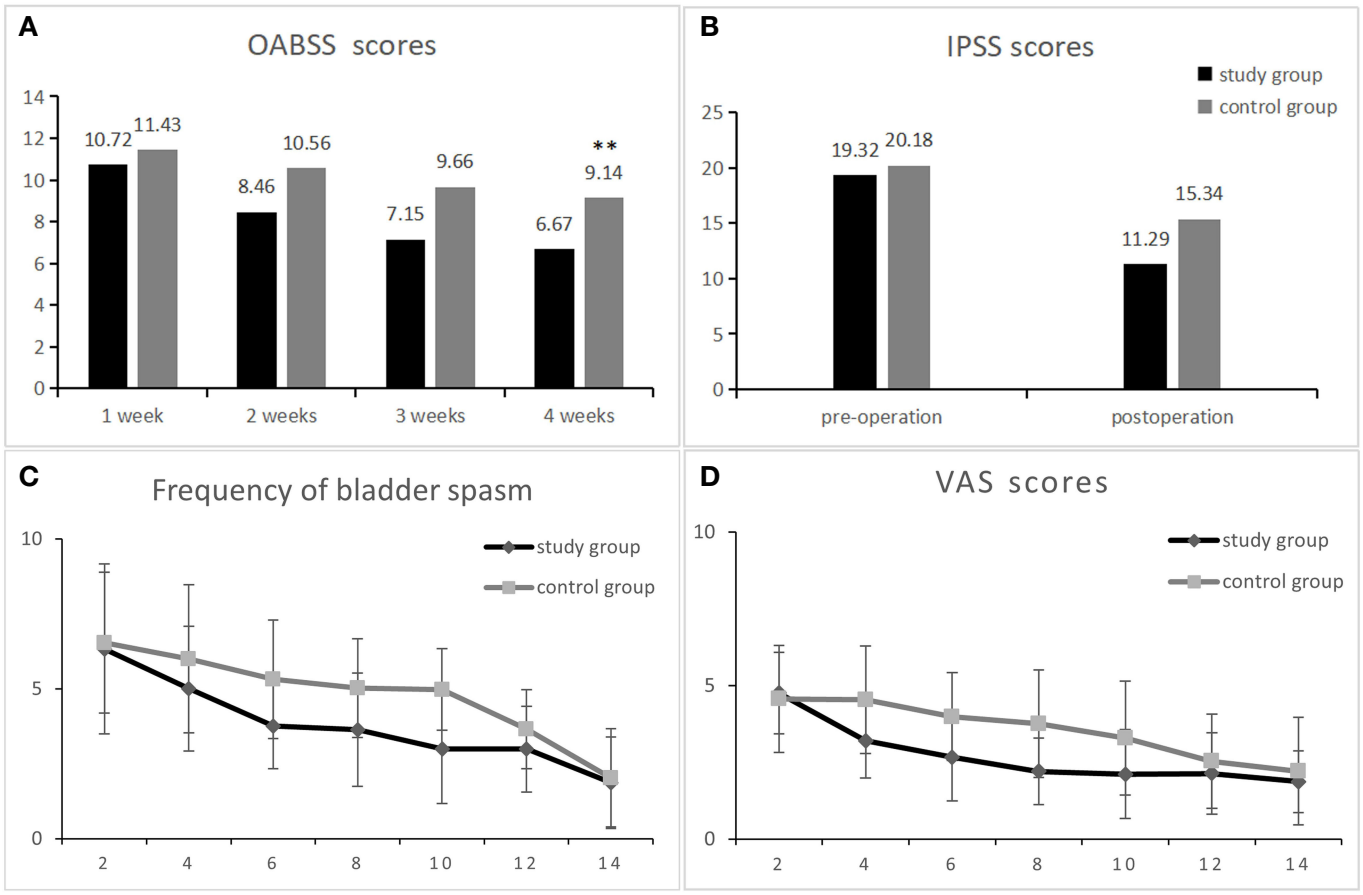
Comparison of data between the research group and the control group after surgery. OABSS scores at 1-4 weeks postoperatively in both groups **(A)**. Comparison of IPSS scores between the research group and the control group from 1 month pre-surgery and 1 month post-surgery **(B)**. The two groups of patients with bladder spasm and visual simulation scores (VAS) 2-14 days after surgery **(C, D)** ** represents a statistically significant difference.

The postoperative rechecked PSA was within the normal range in all patients. As can be seen from **Figure 2**, the change in OAB scores was more significant in the study and control groups after surgery, changing from the first postoperative week (10.72 ± 2.35) and (11.43 ± 2.83) to the fourth postoperative week (6.67 ± 1.06) and (9.14 ± 1.83), and it can be seen that the change in OAB scores was more significant in the study group during drug treatment. The OAB score was significantly lower in the study group compared to the control group at 2, 3, and 4 weeks postoperatively (**Figure 2A**). At week 8 of follow-up, the study group had a significantly lower OABSS score than the control group (5.76 ± 1.39 vs. 7.78 ± 1.53,p<0.01). At the last follow-up, the decrease in OABSS scores was further statistically significant in the study group compared to the control group (4.49 ± 1.35 vs. 5.92 ± 1.45,p<0.01), especially in terms of urinary frequency and incontinence symptoms (0.24 ± 0.43 vs. 1.10 ± 0.61,p<0.01 and 1.58 ± 0.92vs. 1.94 ± 0.81 p<0.05). However, at 12 weeks postoperatively, nocturia and urgency symptoms were not significantly different between the two groups (**Table 2**). This result indicates that the administration of mirabegron in the study group did accelerate the improvement of urinary urgency, nocturia, and urinary incontinence symptoms in patients.

**Table 2:** The comparison of OABSS scores, IPSS scores, and QOL scores during drug treatment and follow-up.

	4 weeks postoperative (end of treatment)			8 weeks postoperative (follow-up)			12 weeks postoperative (follow-up)		
	Study group (n = 55)	Control group (n = 53)	P value	Study group (n = 55)	Control group (n = 53)	P value	Study group (n = 55)	Control group (n = 53)	P value
OABSS total Score	6.67 ± 1.06	9.16 ± 1.61	<0.05	5.76 ± 1.39	7.78 ± 1.53	<0.01	4.49 ± 1.35	5.92 ± 1.45	<0.01
urinary frequency	1.35 ± 0.55	1.59 ± 0.50	<0.01	0.93 ± 0.74	1.31 ± 0.62	<0.01	0.24 ± 0.43	1.10 ± 0.61	<0.01
nocturia	1.75 ± 0.55	2.22 ± 0.78	<0.01	1.67 ± 0.58	1.84 ± 0.70	0.18	1.49 ± 0.74	1.57 ± 0.61	0.56
urgency	1.56 ± 0.50	2.16 ± 0.88	<0.01	1.24 ± 0.43	1.98 ± 0.73	<0.01	1.18 ± 0.39	1.31 ± 0.68	0.23
incontinence	2.14 ± 0.67	3.18 ± 1.01	<0.01	1.93 ± 1.00	2.65 ± 0.91	<0.01	1.58 ± 0.92	1.94 ± 0.81	<0.05
IPSS	11.29 ± 3.21	15.31 ± 4.02	<0.01	8.32 ± 3.96	11.43 ± 3.26	<0.01	7.43 ± 3.12	8.65 ± 2.94	<0.05
QOL	2.40 ± 0.81	3.20 ± 1.00	<0.01	2.05 ± 0.68	1.76 ± 0.76	<0.05	1.55 ± 0.66	1.59 ± 0.75	0.76

There was no significant difference in IPSS scores as well as QOL scores between the study and control groups before surgery, and during the postoperative 1-month drug treatment period, the voiding symptoms and quality of life scores improved in both groups, decreasing from (19.32 ± 3.71 and 20.18 ± 2.93) to (11.29 ± 3.89 and 15.34 ± 3.54, p < 0.01) (**Figure 2B**), indicating that the treatment effect of the study group was better than that of the control group. At week 12 of follow-up, the IPSS continued to decrease in both groups as (7.43 ± 3.12 vs. 8.65 ± 2.94, p < 0.01), as shown in **Table 2**. In addition, **Table 2** shows that postoperative QOL scores decreased in both groups and that patients’ quality of life gradually improved as time progressed.

In addition, our analysis found that both the study group and the control group showed a trend of initial increase and subsequent decrease in OABSS scores. The OABSS scores of patients in the experimental group were higher than the preoperative baseline data from the first to the fourth week after surgery and at the eighth week after surgery, and were statistically different (P < 0.01); however, at 12 weeks after surgery, there was no significant statistical difference in OABSS scores between the experimental group patients and their baseline data before surgery (P > 0.05). In contrast, the OABSS scores of the control group patients within 3 months after surgery were higher than before surgery and showed a statistically significant difference (P < 0.01). Similarly, we analyzed the IPSS and QOL scores of both groups of patients at 4, 8, and 12 weeks after surgery and found that they were higher than their respective baseline data before surgery, and the differences were statistically significant (P < 0.01). These findings indicate that both groups of patients had higher IPSS and QOL scores and lower OABSS scores before surgery, which may be due to urinary symptoms caused mainly by benign prostatic hyperplasia. However, the prostate tissue was removed during surgery, which improved the patients’ urinary frequency and urgency symptoms, but also caused symptoms such as urinary incontinence due to damage to the bladder sphincter, which was the main reason for the higher postoperative OABSS scores.

The number of bladder spasms and the VAS test were recorded every other day after surgery in both groups. There was no significant difference between groups at postoperative days 1-2. At postoperative days 4, 6, 8 and 10, the number of bladder spasms and the degree of pain were significantly higher in the control group than in the experimental group (P < 0.01), and after postoperative day 12, the symptoms gradually improved similarly in both groups (**Figures 2C, D**). **Table 3** shows the indicators of the two groups of patients reviewed at the end of drug therapy. The mean number of days with indwelling drain and catheter was significantly lower in the study group compared with the control group (P < 0.01); in addition, there was no statistical difference in the maximum urinary flow rate at the time of review between the two groups.

**Table 3:** One-month postoperative review of indicators and adverse drug reactions.

	Study group (n = 55)	Control group (n = 53)	P value
Days of catheter placement	8.44 ± 2.04	10.53 ± 2.17	<0.01
Days in drainage tube placement	4.49 ± 0.92	4.37 ± 1.05	0.55
Qmax, ml/s	19.7 ± 8.0	17.6 ± 7.3	0.46
Adverse drug reactions			
Tachycardia	2(3.6%)	0	–
Constipation	3(5.4%)	4(7.5%)	–
Dry mouth	0	2(3.8%)	–

### 3.3 Complications

As shown in **Table 3**, no serious adverse events were observed in either group. In the study group, 2 (3.6%) of 55 patients had tachycardia and 3 (5.4%) had constipation; in the control group, 7 (7.5%) of 53 patients had constipation and 2 (3.8%) had bitter mouth.

## 4 Discussion

Currently, RP remains the treatment of choice for localized prostate cancer. It can significantly improve symptoms of abnormal urination because the surgery removes anatomical bladder resistance (4). Despite the continuous development of surgical methods and techniques, it is inevitable that the anatomical areas around the prostate, such as the triangle, the external urethral sphincter, the bladder neck, and the posterior urethra, are disrupted during surgery, resulting in urinary incontinence and demobilization of the detrusor muscles (10). It has been reported that the incidence of postoperative incontinence after RP ranges from 2.5% to 90% (11–13). Of these, up to one-third of patients are incontinent due to urinary urgency (4, 14). At present, the pathophysiological link between OAB and RP has not been fully elucidated, but most of the literature suggests that the primary contributing factors are detrusor overactivity (DO), urogenic mechanisms, and bladder outlet obstruction (BOO) (4, 11). A study on bladder and urethral function after RP showed that nearly 70.3% of patients were affected by DO after RP, half of whom had varying degrees of OAB symptoms (10). Similarly, a study from New York on the etiology and symptoms of postoperative urinary incontinence after RP suggests that postoperative urinary incontinence is primarily due to sphincter injury and bladder dysfunction (detrusor instability or poor compliance). Urinary incontinence caused by sphincter injury is often associated with increased intra-abdominal pressure, resulting in stress urinary incontinence. Bladder dysfunction is mainly due to intraoperative nerve injury or associated with urinary tract obstruction due to preoperative prostatic lesions and therefore often leads to urinary frequency and urgency as well as urge incontinence after RP (11, 14–16).

The current treatment principles for OAB after RP are the same as those for OAB in general, which mainly include behavioral therapy, pharmacotherapy and surgery (4). Behavioral therapy is primarily used to improve OAB symptoms through bladder training and pelvic floor muscle training. However, there are few data to support this claim; the results of a randomized controlled trial only showed that postoperative incontinence symptoms after RP were significantly improved by preoperative biofeedback combined with postoperative pelvic floor muscle training (17). The main indications for surgical treatment are patients whose urinary control is still not restored 1 year after RP, and surgical procedures include artificial urethral sphincter implantation, ball cavernous suspension, and transurethral injection of fillers (18–20). However, since post-operative RP patients may suffer from erectile dysfunction and retrograde ejaculation symptoms after surgery, which can affect the quality of life and psychological health of patients. Therefore, patients need to be given psychological guidance and comfort during treatment. However, at present, the main method of OAB treatment after RP surgery is pharmacological treatment (21, 22). The efficacy of oral anticholinergics to ameliorate OAB symptoms after RP has been demonstrated in the past few years (13, 23), but the drug is plagued with poor patient compliance due to a large number of side effects. Among them, dry mouth is the most important side effect, followed by possible side effects such as constipation, blurred vision, and cognitive dysfunction (24).

Studies have confirmed the presence of β-AR subtypes (β1, β2 and β3) on bladder forceps and uroepithelial cells, and the key role of β-adrenoceptors in the bladder is to allow smooth muscle relaxation and increased bladder compliance during the filling phase of the voiding cycle. Sympathetic release of metanephrine acting on β3-adrenergic receptors leads to increased levels of intracellular cyclic adenosine monophosphate (cAMP), which induces relaxation of the bladder forcing muscles (25–28). In a study from Emma Mitidieri et al. they experimentally verified that β3-adrenoceptors also act on cystathionine-β-synthase (CSE) in human urinary epithelial cells, resulting in the derivation of more hydrogen sulfide, which in turn leads to an increase in cAMP, thus reinforcing the role of β3-adrenoceptors in regulating the bladder (29). In addition, animal model studies have shown that β3-adrenoceptor agonists can increase bladder volume without altering voiding pressure or residual urine volume. Mirabegron, as the first β3-adrenoceptor agonist approved for the treatment of OAB, can significantly reduce urinary frequency and incontinence in patients with OAB (8, 30, 31). In a large multicenter randomized double-blind study, patients aged 18 years or older with OAB symptoms for ≥3 months were given 50 mg of mirabegron once daily, and the results showed a significant reduction in the frequency of voiding and incontinence and a significant increase in the volume of urine voided per voiding in the experimental group compared to the control group (32). However, there are no corresponding studies evaluating its safety and efficacy on postoperative OAB after RP. In the present study, the experimental group showed significant improvement in the IPSS as well as OABSS scores after RP compared with the control group; in addition, the number of bladder spasms and the degree of pain recorded in the experimental group were significantly lower than those in the control group at 4, 6, 8, and 10 days after surgery. We speculate that the mechanism of action is similar to anticholinergic drugs. The inflammation around the anastomosis after RP and the irritation from the catheter cause bladder irritation symptoms. By inhibiting excessive contraction of the bladder forcing muscle, mirabegron can reduce bladder irritation symptoms and promote healing of the anastomosis, thus reducing the severity of pain reported by the patient. Therefore, it can be demonstrated that mirabegron has a significant effect in relieving the overactivity of the bladder forcing muscles after RP surgery (33–35).

Although this study is a randomized controlled experiment, it has some limitations. The time for follow-up was short, which may have affected the outcome. In addition, we did not test multiple dosage of mirabegron, so we could not know the optimal dose of the drug. However, the treatment results were also significant in our study. In this study, the adverse effects reported by the experimental group receiving 50 mg mirabegron daily were not significantly different from those in the placebo group, and only 2 patients showed symptoms of tachycardia, but it was not serious. In addition, no other serious complications occurred, suggesting that mirabegron has a better safety profile and tolerability than anticholinergic drugs. Most studies have shown that mirabegron has a generally low incidence of cardiovascular adverse events in an older range of subjects (over 60 years of age) and has almost no significant effect on patient heart rate under initial dose conditions (36–38).

In summary, mirabegron can significantly improve symptoms such as urinary frequency, urgency and urge incontinence after RP. Therefore, in the future, it has important therapeutic value in the treatment of postoperative OAB or new-onset OAB for RP.

### 4.1 Figures

Frontiers requires figures to be submitted individually, in the same order as they are referred to in the manuscript. Figures will then be automatically embedded at the bottom of the submitted manuscript. Kindly ensure that each table and figure is mentioned in the text and in numerical order. Figures must be of sufficient resolution for publication. Figures which are not according to the guidelines will cause substantial delay during the production process. Figure legends should be placed at the end of the manuscript. Please see here for full Figure guidelines

### 4.2 Tables

Tables should be inserted at the end of the manuscript. Tables must be provided in an editable format e.g., Word, Excel. Tables provided as jpeg/tiff files will not be accepted. Please note that very large tables (covering several pages) cannot be included in the final PDF for reasons of space. These tables will be published as Supplementary Material on the online article page at the time of acceptance. The author will be notified during the typesetting of the final article if this is the case.

## Data availability statement

The original contributions presented in the study are included in the article/supplementary material. Further inquiries can be directed to the corresponding author.

## Ethics statement

The studies involving human participants were reviewed and approved by Ethics Committee Review Approval of Shanghai Sixth People’s Hospital. The patients/participants provided their written informed consent to participate in this study.

## Author contributions

RY and HX conceived the study. WL drafted the manuscript. WL and YL collected the clinical data and conducted statistical analysis on the data. RY and HX revised the manuscript rigorously. All authors contributed to the article and approved the submitted version.
